# Supplementary material for: Biodegradation Potential of Polyethylene Terephthalate by the Two Insect Gut Symbionts Xanthomonas sp. HY-74 and Bacillus sp. HY-75
Source: Polymers (Basel). 2023 Aug 25;15(17):3546. doi: 10.3390/polym15173546 (PMC10489954; doi:10.3390/polym15173546)
Supplement: Supplementary file 1 [file polymers-15-03546-s001.zip › polymers-2547808-supplementary.pdf]

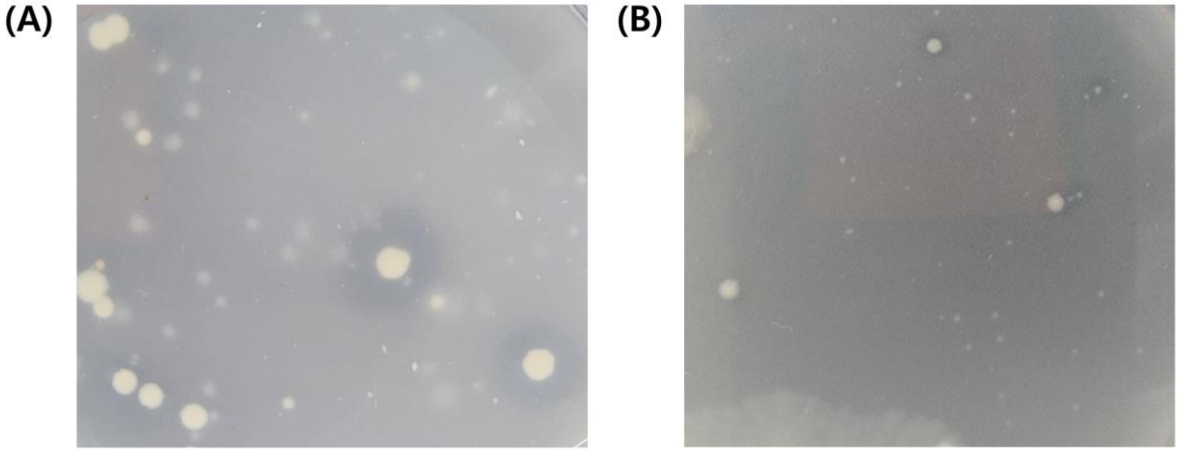

Figure S1. Screening of PET degrading bacteria, *Xanthomonas* sp. HY-74 strain from *Xylocopa appendiculata* (A) and *Bacillus* sp. HY-75 from *Eumenes decorates* (B).
